# Remediation of Cd-Contaminated Soil by Modified Nanoscale Zero-Valent Iron: Role of Plant Root Exudates and Inner Mechanisms

**DOI:** 10.3390/ijerph18115887

**Published:** 2021-05-30

**Authors:** Danlian Huang, Yunhe Yang, Rui Deng, Xiaomin Gong, Wei Zhou, Sha Chen, Bo Li, Guangfu Wang

**Affiliations:** 1 College of Environmental Science and Engineering, Hunan University, Changsha 410082, China; yangyunhe@hnu.edu.cn (Y.Y.); dengrui703@hnu.edu.cn (R.D.); vzhou@hnu.edu.cn (W.Z.); sasa@hnu.edu.cn (S.C.); hndxlibo@hnu.edu.cn (B.L.); wangguangfu1113@163.com (G.W.); 2 Key Laboratory of Environmental Biology and Pollution Control, Hunan University, Ministry of Education, Changsha 410082, China; 3 College of Resources and Environment, Hunan Agricultural University, Changsha 410128, China; gongxm2014@yeah.net

**Keywords:** nanoscale zero-valent iron, rhizosphere, citric acid, cadmium, microbial community

## Abstract

In this study, the role of exogenous root exudates and microorganisms was investigated in the application of modified nanoscale zero-valent iron (nZVI) for the remediation of cadmium (Cd)-contaminated soil. In this experiment, citric acid (CA) was used to simulate root exudates, which were then added to water and soil to simulate the pore water and rhizosphere environment. In detail, the experiment in water demonstrated that low concentration of CA facilitated Cd removal by nZVI, while the high concentration achieved the opposite. Among them, CA can promote the adsorption of Cd not only by direct complexation with heavy metal ions, but also by indirect effect to promote the production of iron hydroxyl oxides which has excellent heavy metal adsorption properties. Additionally, the H^+^ dissociated from CA posed a great influence on Cd removal. The situation in soil was similar to that in water, where low concentrations of CA contributed to the immobilization of Cd by nZVI, while high concentrations promoted the desorption of Cd and the generation of CA–Cd complexes which facilitated the uptake of Cd by plants. As the reaction progressed, the soil pH and cation exchange capacity (CEC) increased, while organic matter (OM) decreased. Meanwhile, the soil microbial community structure and diversity were investigated by high-throughput sequencing after incubation with CA and nZVI. It was found that a high concentration of CA was not conducive to the growth of microorganisms, while CMC had the effect of alleviating the biological toxicity of nZVI.

## 1. Introduction

Environmental issues caused by the heavy metal contamination of soils are increasingly becoming a global threat due to their harmful effects on soil ecosystems and human health [1,2]. Among these heavy metals, cadmium (Cd) is one of the most hazardous heavy metals because of its high toxicity and bioavailability [3]. It is extremely toxic for living organisms accumulating Cd with high mobility and its neurotoxic, mutagenic and carcinogenic nature [4]. At higher Cd concentrations, it can also induce oxidative stress in plants by stimulating the production of more reactive oxygen species (ROS) and lipid peroxidation, and ultimately lead to death. Human activities are the main causes of soil Cd pollution including the application of chemical fertilizer and sludge, wastewater irrigation, mining and smelting, dry and wet atmospheric deposition and solid waste disposal [5]. Therefore, it is urgent to solve or alleviate the problem of Cd pollution in the soil.

In recent years, the application of nano zero-valent iron (nZVI) in groundwater and soil remediation has been well reported due to its superior properties including a large specific surface area and high reduction capacity, which enable it to remove heavy metals efficiently [6]. In Cd-contaminated soils, the alteration products (iron oxides or hydroxides) produced by the oxidation of nZVI accelerate the immobilization of Cd through adsorption, morphological changes, surface precipitation and co-precipitation [7]. However, one obvious problem is that nZVI tends to aggregate rapidly due to attractive magnetic forces, which greatly reduces its responsiveness to contaminants [8]. Meanwhile, with the widespread introduction of nZVI into the environment, the potential damage to the ecosystem has attracted considerable attention due to the high surface activity and bioavailability of nZVI [9,10]. For instance, nZVI and ferrous ion can inactivate MS2 coliphage in aqueous solution regardless of whether they are under anaerobic or aerobic conditions [11]; this showed strong toxicity to Typha grown hydroponically at higher concentrations (>200 mg/L) [12]. In addition, it was proven that the high concentration of starch-stabilized nZVI (S-nZVI) can inhibit ramie growth and aggravate the oxidative damage to plants [1]. In order to improve the stability and mobility of nZVI and alleviate its toxicity, various modifications have been made, among which carboxymethyl cellulose (CMC)-stabilized nZVI (CMC-nZVI) is considered one of the most successful combinations, based on not only lab experiments but also field tests [10,13,14,15,16]. CMC is a kind of polyelectrolytes with both carboxyl and hydroxyl groups [17]. CMC as a stabilizer can generate electrostatic and steric repulsion forces between the nanoparticle surfaces, thus preventing nZVI particles from aggregating due to magnetic and van der Waals attractions [18]. As for the toxicity of CMC-coated nZVI, the polymer coating on nZVI can act as a barrier between microbial cell walls and the highly reactive nZVI surface, thereby mitigating oxidative stress [10]. According to Zhou et al., CMC-nZVI was supposed to cause minimized oxidative stress response and slow damage to cell wall integrity, which leads to its less toxic effect on *Agrobacterium* sp. than bare nZVI [19,20]. In addition, the immobilization efficiency of CMC-nZVI for Cd(II) in sediments was better than that of nZVI without stabilization [21]. Thus, it is appropriate to choose CMC-nZVI as the experimental subject of this study.

The compounds in root exudates can be classified as either high molecular weight or low molecular weight materials [22]. Among these, low molecular weight organic acids (LMWOAs) such as malic, oxalic, acetic, fumaric and citric acid (CA) are negatively charged anions that are capable of forming stable complexes with bioavailable Cd^2+^ to influence plant Cd uptake [23]. For example, Sana Ehsan et al. reported that CA attenuated Cd toxicity by reducing malondialdehyde and H_2_O_2_ levels to reduce oxidative stress and enhancing antioxidant enzyme activity under Cd stress [24]. Hassen et al. reported that CA promoted the uptake of Cr by *Pseudomonas aeruginosa* and Cu by *Bacillus thuringiensis* [25]. These studies all suggest that LMWOAs play an important role in the rhizosphere environment stressed by heavy metals. However, it is very difficult to find the basis for a certain kind of organic acid secreted by all plants to play a key role under heavy metal stress from previous studies. The secretion of CA in the root exudates of certain plants is more significant when they are exposed to environmental stress [26,27]. At the same time, CA also has a wider range of applications, such as food processing [28], material synthesis [29], and soil leaching [30]. Additionally, compared to other organic acids, its cost is lower. Thus, CA was selected in this study for the aforementioned reasons.

The main objectives of the present study were to “(ⅰ) simulate the soil pore water environment to investigate the effect of CA at different concentrations and pH on Cd(Ⅱ) removal from water by CMC-nZVI; (ⅱ) simulate rhizosphere soil contaminated with Cd, containing different concentrations of CA and nZVI or CMC-nZVI, and investigate the changes in Cd morphology, pH, OM and CEC in the treatment groups; (ⅲ) investigate the response mechanisms of the microorganisms in rhizosphere.”

## 2. Materials and Methods

### 2.1. Soil Characterization and Preparation

The soil was collected from Mt. Yuelu in Hunan province in southern China, which contained a high organic mass fraction and a pH between 4 and 4.5, making it a strongly acidic soil. Then, the soil was contaminated with cadmium nitrate (Cd(NO_3_)_2_) solution for two months [31,32]. After that, the treated soil was air-dried at room temperature and crushed and sieved through a 100-mesh sieve for subsequent experiments. The total Cd content in the soil was determined at about 50 mg/kg.

### 2.2. Nanoparticles Synthesis and Characterization

The detailed process of nZVI and CMC-nZVI preparation and characterization were displayed in Text S1 of the Supplementary Materials [3,6,21].

### 2.3. Experimental Design of Simulated Pore Water and Rhizosphere Environment

Contaminated soil pore water was simulated with the 50 mg/L Cd(NO_3_)_2_ solution and the pH was adjusted to 4.0 with hydrochloric acid (HCl). In order to investigate the effect of different concentrations of CA on the removal of Cd(Ⅱ) from water by modified nZVI, 15 mg of CnZVI-6 (0.2 wt% CMC-nZVI) was added to 30 mL of the contaminated solution, and then the different concentrations of CA (0, 5, 15, 30 mg) were added, and the Cd(Ⅱ) concentrations in the solutions were determined by atomic absorption spectrometry after 6 h of shaking in a water bath. To eliminate the effect of hydrogen ions on the removal of Cd, an equal amount of modified nZVI was added to the solution which was adjusted to different pH (4, 5, 7) by adding HCl or CA as a contrast. Additionally, their products were analyzed by X-ray photoelectron spectroscopy (XPS) and XRD.

In the experiment of simulating the surrounding rhizosphere, 20 g of treated soil was taken into a 50 mL centrifuge tube for each sample. According to previous studies of Wan and Gong et al. [1,33], there were less phytotoxicity to plants and a better metal stabilization effect when the content of nZVI was 500 mg/kg in the soil. The soil samples were prepared in three categories: (1) treatments with the addition of CA (0, 50, 250, 500 mg/kg), represented by 0, 1, 5, 10, respectively; (2) treatments with addition of CA (0, 50, 250, 500 mg/kg) + nZVI (500 mg/kg) were represented by N0, N1, N5, N10, respectively; (3) treatments with addition of CA (0, 50, 250, 500 mg/kg) + CnZVI-6 (500 mg/kg) were represented by C0, C1, C5, C10, respectively. Specifically, 10 mg of nZVI or CnZVI-6 was loaded into the centrifuge tube and the tube was shaken vigorously for 10 min to mix the materials and soil samples evenly. Then, 5 mL of CA solution with different concentrations (0, 0.2, 1.0, 2.0 mg/L) was poured into each tube and fully contacted with the soil by vigorously shaking the tube again. Three parallel samples were established for each group. Afterwards, the samples were placed in a constant temperature and humidity incubator, and the parameters including the concentration of each form of Cd(Ⅱ), pH, OM, and CEC were measured periodically. The impact on soil microbial communities was assessed after an incubation period of one month.

### 2.4. Fractions of Cd in the Soil Samples

The speciations of Cd in the samples were measured by the modified sequential extraction method of the European Community Bureau of Reference (BCR). The extractions were divided into four steps corresponding to four fractions separately. Acid extractable/exchangeable fraction: acetic acid was added to the samples, and the samples were centrifuged to take the supernatant for a measurement for after a period of reaction. Reducible fraction: hydroxylammonium chloride was added to the residue of step 1. The following steps were the same as above. Oxidizable fraction: the residue from step 2 was treated by hydrogen peroxide, followed by adding ammonium acetate to the residue. Residual fraction: the residue of step 3 was digested with aqua regia and hydrofluoric acid. The detailed process is displayed in Text S2 of Supplementary Materials [34].

### 2.5. Methods for the Detection of Soil Physico–Chemical Properties

Soil pH was measured by diffusing 1 g of soil into water at a ratio of 1:5 *w*/*w* of soil to water. The potassium dichromate volumetric method–dilution heating method was used for determination of soil OM content. The CEC value was determined by barium chloride-sulfuric acid enhanced exchange method. The detailed process is displayed in Text S3 of the Supplementary Materials [35].

### 2.6. High-Throughput Sequencing

Five samples, Z0 (control group), nZVI_0 (nZVI only), CnZVI_0 (CnZVI only), CnZVI_1 (CnZVI + 50 mg/L CA), and CnZVI_10 (CnZVI + 500 mg/L CA), were preincubated for 30 days and then outsourced to Shanghai Majorbio Bio-pharm Technology Co., Ltd., Shanghai, China for the high-throughput sequencing. The final data were obtained from the free online platform of Majorbio Cloud Platform (www.majorbio.com (accessed on 21 December 2020)). The detailed method of high-throughput sequencing is shown in Text S4 of Supplementary Materials [36].

### 2.7. Statistical Analysis

The data shown in this study are mean values ± standard error (SE) of three replicated treatments. Significant differences were determined by one-way ANOVA according to the Tukey test in SPSS v26.0 (IBM Corp., Armonk, NY, USA). Correlation analysis was performed using Software R v3.6.3 (RStudio, Vienna, Austria). principal component analysis (PCA) about treatments containing a different microbial community composition on OUT level was performed by Majorbio Cloud Platform (www.majorbio.com (accessed on 4 January 2021)). The platform took the two eigenvalues of principal components (PC1, PC2, respectively) that could best reflect the difference between the samples automatically. In addition, CMC and CA contents were considered as two environment factors and presented by arrows in the PCA analysis diagram.

## 3. Results and Discussion

### 3.1. Materials Characterization

The morphologies of bare nZVI and CnZVI-6 were examined by SEM analysis. For bare nZVI, the SEM images are shown in Figure S1A of the Supplementary Materials and demonstrated that the nZVI particles were irregularly clustered together, and mostly appeared as short chain-like structures or amorphous groups. As shown in Figure S1B, aggregates formed by the aggregation of nanoparticles were much larger than the individual particles, which severely affected the performance of the materials. SEM images were also taken for CnZVI-6, from which small sphere-like particles with a small size (D < 120 nm) and low agglomeration can be clearly observed (Figure S1C,D). This was ascribed to the magnetic particles encapsulated by CMC with a negative surface charge resulting in a strong electrostatic repulsion between the stabilized particles, which decreased the agglomeration of magnetic particles. It was confirmed based on the SEM images (Figure S1) that the CMC was successfully coated onto the nanoparticles, thus improving the aggregation-prone nature of nZVI.

The phase composition and crystals of the bare nZVI and CnZVI-6 particles were identified by XRD analysis (Figure S2 of Supplementary Materials). There are significant peaks at 2θ = 44.8° assigned to Fe^0^ in both the nZVI and CnZVI-6 XRD patterns [21]. The only difference is that the peak of CnZVI-6 is obviously smoother than that of nZVI, which might correlate with the decreased crystallinity or a disturbance from the CMC macromolecules on the iron surface. This also provides evidence that the modification of CMC affects the performance of simple nZVI nanoparticles.

### 3.2. Effect of CA Concentration on Removal of Cd(Ⅱ) from Aqueous Phase

**Figure 1:**
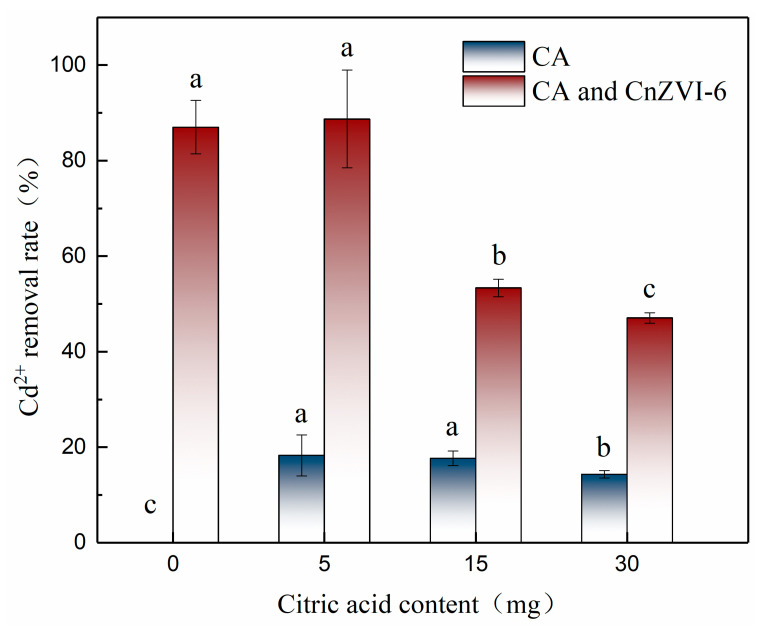
The removal efficiency of Cd(II) by CA with different concentrations (0, 5, 15, 30 mg) in the presence or absence of 15 mg CnZVI-6, respectively, in the solution (mean ± SE, *n* = 3). Different letters indicate significant differences among the CA groups with or without CnZVI-6 treatment at the *p* < 0.05 level according to Tukey test.

Due to the complex rhizosphere environment, experiments in Cd solution were first carried out in order to investigate the underlying principles of the influence of CA on the interaction between CnZVI-6 and Cd ions. The CA with different concentrations (0, 5, 15, 30 mg) was added to 30 mL of 50 mg/L cadmium solution in the presence or absence of 15 mg CnZVI-6, respectively. The removal efficiency of Cd(II) in the solution was shown in Figure 1. One can see that the removal efficiency of CA alone for Cd(II) in water decreased with an increasing concentration, with the maximum value of 18.28% at a CA content of 5 mg. This phenomenon indicates that there is a certain chelation between CA and Cd ions, which reduces the concentration of free Cd^2+^. At the same time, the concentration of hydrogen ions increases with the increase in the CA concentration, which will make the competition between hydrogen ions and Cd^2+^ more intense, thus reducing the stability of the chelate formed by CA and Cd^2+^. On the other hand, a higher concentration of hydrogen ions can also decompose the iron oxides produced after the oxidation of modified nZVI and thus affect the adsorption of Cd on the materials. From Figure 1, we found that the removal rate was about 87% when only CnZVI-6 was used to remove free Cd^2+^ from the solution. However, the removal effect changed significantly when CA was added to the solution with different concentrations. When the content of CA in the solution was 5 mg, the removal efficiency of Cd^2+^ was slightly increased to 88.73%, while the removal efficiency sharply decreased to 53.34% and 47.07% when the content was 15 mg and 30 mg, respectively. The results showed that CA promoted the removal of Cd^2+^ by 0.2 wt% CMC-nZVI at low concentrations, while it inhibited the removal of Cd^2+^ at high concentrations, and the inhibitory effect increased with increasing concentrations.

### 3.3. Effect of CA on the Removal of Cd^2+^ from Water by Modified nZVI under Different pH

**Figure 2:**
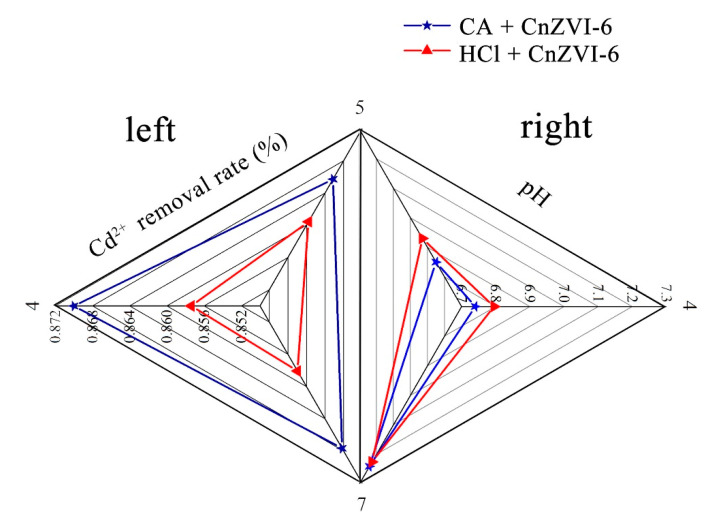
The Cd^2+^ removal efficiency with (★) CA or HCl (▲) at different pH 4, 5 and 7 (**left**) and the pH change after the reaction (**right**).

In order to exclude the influence of hydrogen ions, we conducted a hydrogen ion shielding experiment, i.e., using CA to adjust the pH of the solution to 4, 5 and 7, and then kept the other reaction conditions unchanged using HCl instead of CA for comparison, in order to investigate the effect of CA on the removal effect without the influence of hydrogen ions. Figure 2 shows the Cd^2+^ removal efficiency with CA or HCl at a different pH (4, 5, 7) on the left side and the other side shows the pH change after the reaction. From the left side of Figure 2, it is obvious that the addition of CA contributes to enhance the removal efficiency compared to the HCl control group, and the highest removal rate was up to 87% at pH 4. This result further confirms that the metal chelation produced by CA is greater than the resolution of H^+^ at a certain concentration, thereby facilitating the removal of Cd^2+^ [37]. As for pH, the pH of all the samples increased after the reaction as shown in the right part of Figure 2, and the pH of the HCl control group was slightly higher than that of the CA group [38]. This is because the pH increased with the oxidation of Fe^0^ in the solution, and since HCl is a strong electrolyte and CA is a weak electrolyte, the concentration of CA is higher at the same pH. Therefore, as the pH increased, CA would release more H^+^, making the pH slightly lower than that of the HCl control group.

### 3.4. The Changes of Reaction Products in the Water System

**Figure 3:**
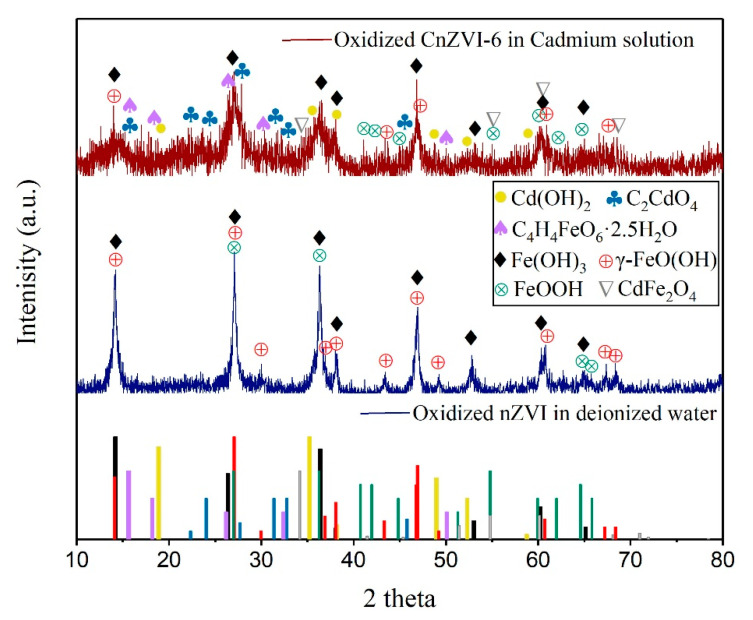
XRD analysis of the composition evolution of the bare nZVI in deionized water (blue line) and CnZVI-6 particles in cadmium solution (red line).

In order to further explore the specific reaction mechanism, the composition evolution of the bare nZVI in deionized water and CnZVI-6 particles in Cd solution were investigated by XRD analysis (Figure 3). The blue XRD spectrum in Figure 3 showed the predominant crystalline forms of iron oxides produced by the oxidation of nZVI in deionized water. The main components of iron oxides are Fe(OH)_3_ (peaks at 2θ = 26.4°, 36.4°, 38.0°, 46.9°, 53.0°, 60.2° and 65.1°) and FeOOH (peaks at 2θ = 36.2°, 40.7°, 41.9°, 44.8°, 51.3°–64.6°, 65.8°), and FeOOH is dominated by lepidocrocite (γ-FeOOH) with peaks at 2θ of 38.1°, 43.3°, 46.8°–60.7°, 67.2°, 68.4°. Based on the similar standard oxidation reduction potentials of Cd (E^0^ = −0.40 V) and Fe (E^0^ = −0.41 V) [39], the likelihood of Cd(II) being reduced to Cd(0) on nZVI is low. Thus, it can be concluded that the mechanisms of Cd removal by nZVI are likely physical sorption, chemisorption or surface complex formation, instead of direct reduction [40]. For the situation in this experiment, the sorption and complexation mechanisms of Cd on the FeOOH polymorphs have been investigated comprehensively in recent years [41], which can be explained in terms of the negative surface charge on iron oxides when surface functional groups deprotonate as pH rises during the oxidation of nZVI to FeOOH polymorphs [42]. The inner sphere complexation between Cd and FeOOH is generated via edge-sharing and corner-sharing adsorption to Cd provided by the surface oxide layer, where the resulting Cd–Fe and Cd–O linkages can be observed and confirmed by the appearance of the CdFe_2_O_4_ (▽) in the part of Figure 3 that shows the oxidized CMC-nZVI in Cd solution. The elevated pH also causes some Cd ions to combine with hydroxide ions to form Cd(OH)_2_ precipitates on the surface of the material. The surface reactions of Cd removal by nZVI may be described by the following equations:

(1) $${\mathrm{Fe}}^{0}{+2H}_{2}O\;\to {\;\mathrm{Fe}}^{2+}{+H}_{2}{+2\mathrm{OH}}^{-}$$

(2) $${\mathrm{Fe}}^{2+}{+O}_{2}{+2H}_{2}O\;\to {\;\mathrm{Fe}}^{3+}{+4\mathrm{OH}}^{-}$$

(3) $${\mathrm{Fe}}^{3+}{+2H}_{2}O\;\to {\;\mathrm{FeOOH}+3H}^{+}$$

(4) $$\equiv {\mathrm{FeO}}^{-}{+\mathrm{Cd}}^{2+}\;\to \;\equiv {\mathrm{FeOCd}}^{+}$$

(5) $$\equiv {\mathrm{FeOH}+\mathrm{Cd}}^{2+}{+H}_{2}O\;\to \;\equiv {\mathrm{FeOCdOH}+2H}^{+}$$

(6) $${\mathrm{Cd}}^{2+}{+2\mathrm{OH}}^{-}\;\to \;\mathrm{Cd}{\left(\mathrm{OH}\right)}_{2}$$

Moreover, the hydroxyl and carboxyl groups that make up the structure of CMC make it possible to form chelates with Cd and oxidized nZVI, attributed to the formation of C_2_CdO_4_ and C_4_H_4_FeO_6_·2.5H_2_O crystal structures (Figure 3). The chelation reaction involving CMC can be expressed by the following equation:

$${\left[C_{6}H_{7}O_{2}{\left(\mathrm{OH}\right)}_{2}{\mathrm{OCH}}_{2}{\mathrm{COO}}^{-}\right]}_{n}{+\mathrm{xFeOCd}}^{+}{+y\;\mathrm{Cd}}^{2+}{+\mathrm{zFe}}^{2+}\;\to {\;\mathrm{Cd}}_{y}{\mathrm{Fe}}_{z}{\left(\mathrm{FeOCd}\right)}_{x}{\left[C_{6}H_{7}O_{2}{\left(\mathrm{OH}\right)}_{2}{\mathrm{OCH}}_{2}\mathrm{COO}\right]}_{n}$$

(7) x+2y+2z=n

**Figure 4:**
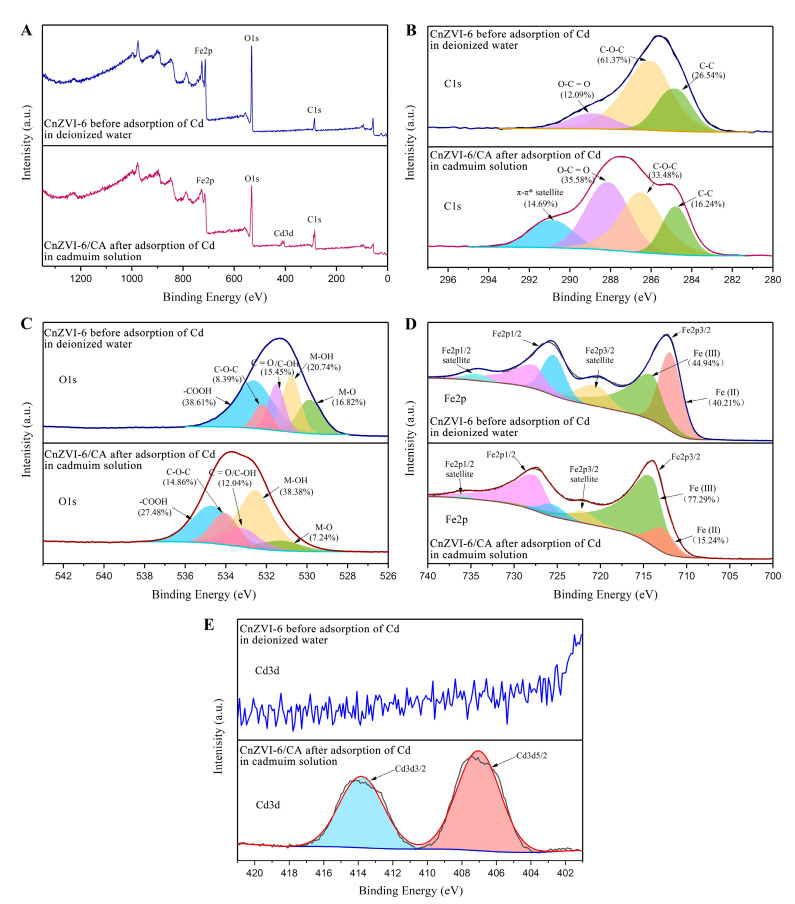
XPS spectra of CnZVI-6 before the adsorption of Cd in deionized water (blue) and CnZVI-6/CA after adsorption of Cd in cadmium solution (red): (**A**) full survey of XPS spectra; (**B**) C 1s; (**C**) O 1s; (**D**) Fe 2p; and (**E**) Cd 3d.

XPS was used to characterize the chemical composition and electronic structure of elements in CnZVI-6 before and after Cd(II) adsorption with CA. The XPS survey spectra in Figure 4A show the presence of C 1s, O 1s, Fe 2p, and Cd 3d. As displayed in Figure 4A,E, the Cd3d region was only present in the form of a divalent at a peak of 407.04 eV compared with the blank contrast sample, indicating that no reduction of Cd occurs during its adsorption onto the adsorbent. The C 1s spectra in Figure 4B was deconvoluted into three peaks at 284.8, 286.0 and 288.9 eV, which were assigned to the C–C, C–O–C and O–C=O functional groups, respectively. These functional groups can also indicate the presence of CMC in the materials. Moreover, the area ratios of O–C=O increased from 12.09% to 35.58% after the addition of CA and Cd(II) adsorption by CnZVI-6. It is likely that the ternary and carboxyl-rich CA increased the content of O–C=O functional groups in the products when it was involved in the reaction and bound to the adsorbent. As presented in Figure 4C, the O 1s spectrum composed of five deconvoluted peaks at 529.9, 530.8, 531.6, 532.2 and 532.7 eV were ascribed to the existence of metal oxide (M-O), hydroxyl bonded to metal (M–OH) and the oxygen content in the form of organic carbon (C=O/C–OH, C–O–C and -COOH), respectively. Moreover, the area ratios of C–OH and -COOH decreased from 15.45% and 38.61% to 12.04% and 27.48% after Cd(II) adsorption by CnZVI-6 with CA added. However, the total area ratios of M–OH and M–O increased from 37.56% to 45.62% after the reaction. The increase in oxygen in metal oxide and hydroxide was mainly attributed to the formation of Cd–O and Cd–OH groups. Additionally, the reduction of the typical coordination groups C–OH and -COOH might ascribe to the complexation or chelation with Cd(II). In addition, the binding energy of oxygen-containing functional groups increased after Cd(II) adsorption by CnZVI-6 with CA. These changes might originate from the chelation between the oxygen atoms and heavy metal ions, leading to its electron density decreased. Therefore, the difference in the interaction between heavy metal ions and oxygen-functional groups in the adsorption process was triumphantly revealed, which was consistent with the analysis of XRD. The Fe 2p spectra in Figure 4D exhibited two double peaks, namely Fe(II) at 711.9 eV (Fe2p3/2) and 725.4 eV (Fe2p1/2) and Fe(Ⅲ) at 714 eV (Fe2p3/2) and 727.6 eV (Fe2p1/2). After the addition of CA and loading with Cd(II) ions, the content of divalent iron decreased from 40.21% to 15.24%, while the content of trivalent iron increased from 44.94% to 77.29%. The reason for this change can be traced back to the reactions that occur in the solution. The Equation (8) is derived from the combination of Equations (2) and (3):

(8) $${4\mathrm{Fe}}^{2+}{+H}_{2}{O+O}_{2}\;\to {\;3\mathrm{Fe}}^{3+}{+\mathrm{FeOOH}+\mathrm{OH}}^{-}$$

The addition of CA increases the concentration of free hydrogen ions in the solution, causing the above reaction to proceed in a positive direction, i.e., promoting the formation of ferric ions and iron hydroxyl oxides. Therefore, CA can promote the adsorption of Cd(II) not only by direct complexation with heavy metal ions, but also by an indirect effect to promote the production of iron hydroxyl oxides, which has excellent heavy metal adsorption properties.

### 3.5. Distribution of Cd Speciation in Soil Samples

**Figure 5:**
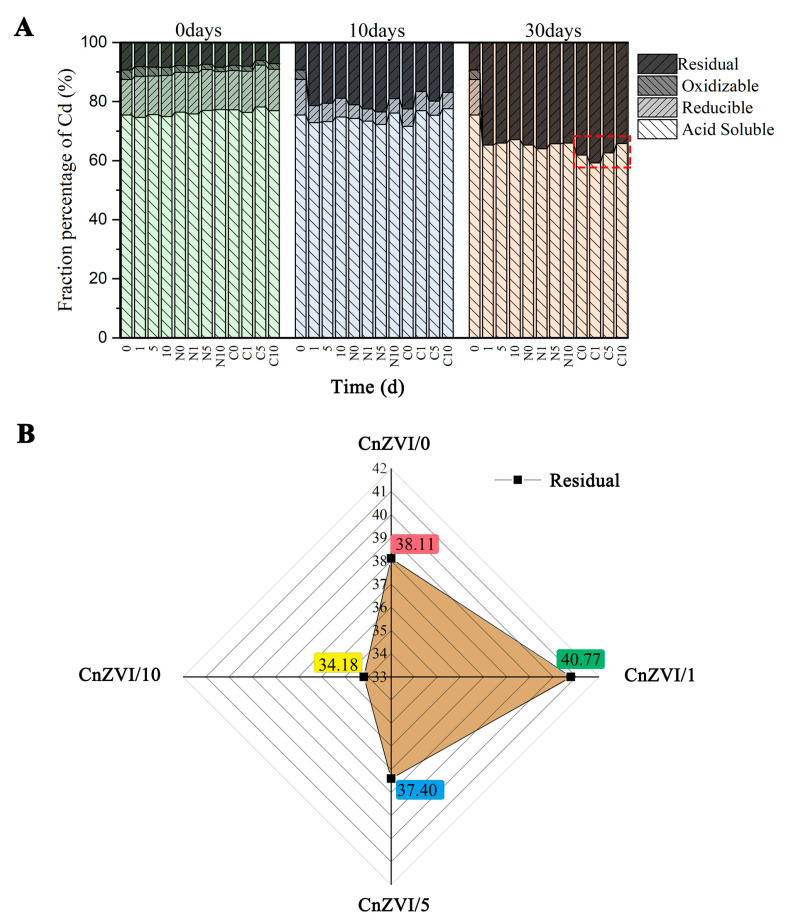
(**A**) The fractioning of Cd was tested as the incubation proceeded for 30 days. (**B**) The residual fraction percentage for four CnZVI groups after 30 days, where CnZVI/0 corresponds to CnZVI-6 with 0 mg/kg CA, CnZVI/1 corresponds to CnZVI-6 with 50 mg/kg CA, CnZVI/5 corresponds to CnZVI-6 with 250 mg/kg CA, and CnZVI/10 corresponds to CnZVI-6 with 500 mg/kg CA.

According to existing reports, nZVI can stabilize soil heavy metals and promote phytoextraction in phytoremediation [1,21]. Generally, the stability of heavy metals is opposite to that of bioavailability. Therefore, there must be some factor behind this contradictory phenomenon that plays a key role. The most significant distinction is the difference between rhizosphere soil and bulk soil [43]. To investigate the effect of nanoparticles and different concentrations of root secretions on the changes of metal distribution, fractionations of Cd in the soil were determined by the modified BCR sequential extraction method for 30 days (Figure 5). In general, the stability of heavy metal precipitates follows this order: acid soluble fraction < reducible fraction < oxidizable fraction < residual fraction. From Figure 5A, it was concluded that the content of acid soluble Cd in the original soil is the highest, accounting for about 75%. With the passage of time, the oxidation fraction and the reduced fraction significantly changed to the residue fraction, while the acid soluble fraction also changed slowly. Observing the data on the 30th day, it can be seen that the groups with CnZVI-6 added accounted for the largest proportion of residual fraction. As displayed in Figure 5B, the maximum residual state ratio was 40.77% with CnZVI/1, while the minimum ratio was 34.18% with CnZVI/10. These results also indicate that CA can promote the stable transition of free Cd at a low level and inhibit it at a high level. Therefore, it can be concluded that when plants secrete low-concentration LMWOAs, they promote the fixation of Cd by nZVI; when the concentration of organic acids is high, the fixation of Cd by nZVI is reduced due to the increase in free hydrogen ions. However, in addition to the fixation effect of nZVI, the free LMWOAs can promote the absorption of Cd by plants, reduce the oxidative stress of free Cd to plants, and enhance the antioxidant capacity of plants [24,44,45]. Additionally, as nZVI ages in the soil, it will increase the alkalinity of the soil, which may induce plants to secrete more organic acids and thereby maintaining a stable pH in the rhizosphere environment.

### 3.6. Changes in Soil Properties

**Figure 6:**
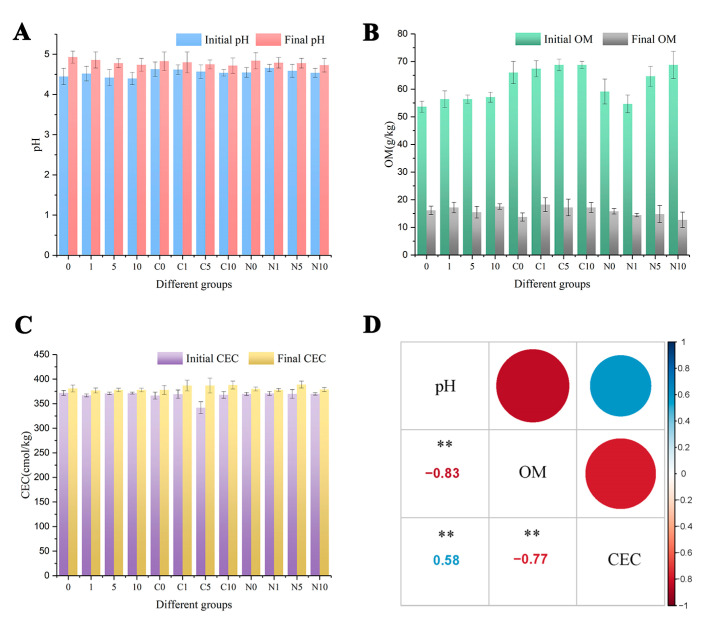
(**A**) The changes in soil pH before and after incubation. (**B**) The changes of OM in the soil. (**C**) The changes of CEC under different treatments before and after incubation. All values represent the mean ± SE, *n* = 3. The one-way ANOVA indicates the significant differences at *p* < 0.05 according to Tukey test. (**D**) The correlation among pH, OM and CEC. “**” means that the correlation at the 0.01 level is significant.

In controlling the heavy metal transfer behavior in soils, pH is a key parameter due to the serious ligand competition between H^+^ and dissolved metals (e.g., OH^−^, CO_3_^2−^, SO_4_^2−^, Cl^−^, S^2−^ and phosphates). Since the soil of Yuelu Mountain is a strongly acidic soil, the Cd in the soil sample is mostly in free form before the reaction (Figure 5A) [46]. From Figure 6A, we can see that the initial soil pH with nZVI (N0, N1, N5, N10) or CnZVI-6 (C0, C1, C5, C10) was higher than the samples in which only CA was added (0, 1, 5, 10), and it was probably due to the oxidation of nZVI in the soil under acidic conditions. Additionally, after 30 days incubation, pH rises to approximately 4.8–4.9 when the initial solution was 4.5–4.6. The soil pH rise was attributed to the release of OH^−^ ions from the oxidation of nZVI and the reaction of this phenomenon is shown in Equation (9):

(9) $${2\mathrm{Fe}}^{0}{+O}_{2\;}{+2H}_{2}O\;\to {\;2\mathrm{Fe}}^{2+}{+4\mathrm{OH}}^{-}$$

The critical pH value for Cd^2+^ hydrolysis (formation of Cd(OH)^+^ and Cd_2_(OH)^3+^) and precipitation (Cd(OH)_2_) is ≥8.0 [47]. The mechanisms of Cd removal by nZVI in soil are likely physical sorption, chemisorption or surface complex formation instead of immobilization through deposition on the surface of iron (oxyhydro) oxide.

In soil ecosystems, OM contains a large number of oxygen-containing functional groups and other functional groups which have a strong complexing and enrichment ability for heavy metals [48,49]. Thus, OM contents in treated samples were detected and the summary was shown in Figure 6B. From the data of initial OM, the OM content of the treatment groups was higher than that of the control group (0), which may be due to the addition of CMC and CA [10]. With the degradation of OM in the soil by microorganisms continued for one month, the contents of OM significantly decreased and became stable. As can be seen from the final content of OM, the remaining OM is basically the same in all treatment groups, which also indicates that the OM in the soil has reached a stable state. The high content of OM (50–70 g/kg) in the soil may explain the observed negligible effect of nZVI and CA on soil microbial properties [50].

CEC was one of the main soil properties controlling Cd retention behavior and negatively correlated with extractable Cd in soil [51]. From Figure 6C, we can see that the CEC value of the soil was very high, around 360–377 cmol/kg, and the values increased as the reaction progressed. The correlation among pH, OM and CEC was shown in Figure 6D. Therefore, from Figure 6D, we can see that OM was negatively correlated with pH and CEC, and CEC was in positive correlation with pH. For the soil environment in this experiment, the oxidation of nZVI, the metabolism of soil microorganisms and the transformation of Cd forms are important factors influencing the soil physicochemical properties OM, pH and CEC. Specifically, with the oxidation of nZVI and the metabolism of microorganisms, the OM content in the soil gradually decreased and the pH gradually increased [21], while a large number of active functional groups (e.g., -COOH, -OH) in OM could also ionize H^+^ and thus lower the soil pH [52]. In addition, with the oxidation of nZVI, iron oxides increased and the pH rose, thus facilitating the transition of Cd to the stable state, which was also reflected in the increase in the CEC value.

### 3.7. Shift of Bacterial Community Composition and Structure under the Experimental Conditions

**Figure 7:**
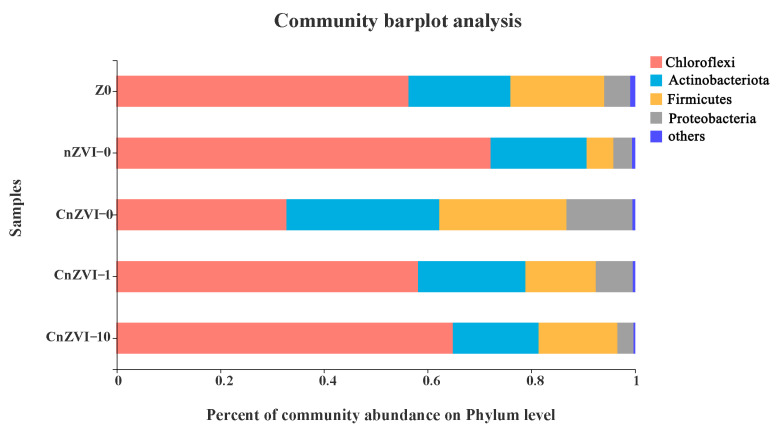
Relative abundance of bacterial 16S rRNA gene at the phylum level.

The relative abundances of the bacterial 16S rRNA gene at the phylum level were observed to study the affiliated composition of the microbial communities (Figure 7). The microbial communities mainly include the *Chloroflexi, Actinomycetes, Firmicutes* and *Proteobacteria*. Many studies have reported that some *Actinomycetes* found in ground water are classified as Fe(Ⅲ)-reducing bacteria. The content of *Actinomycetes* in the CnZVI-0 group is significantly higher than that in the control group Z0, while nZVI-0, CnZVI-1 and CnZVI-10 groups did not increase, which may be due to the biological toxicity of nZVI and the acidity of CA inhibiting the growth of *Actinomycetes* [53,54]. The *Chloroflexi* has better survivability than other phyla under severe conditions, which also shows that the CnZVI-0 group has the least biological toxicity [55]. The *Proteobacteria* of the CnZVI-0 and CnZVI-1 groups are significantly increased compared with the control group [56,57]. This is because CMC, as an external carbon source, provides more nutrients for the growth of microorganisms, whereas the levels of *Firmicutes* and *Proteobacteria* are reduced in both the nZVI and CnZVI-10 groups, which may be related to the biological toxicity of nZVI and the acidic inhibitory effect of high CA concentrations.

**Figure 8:**
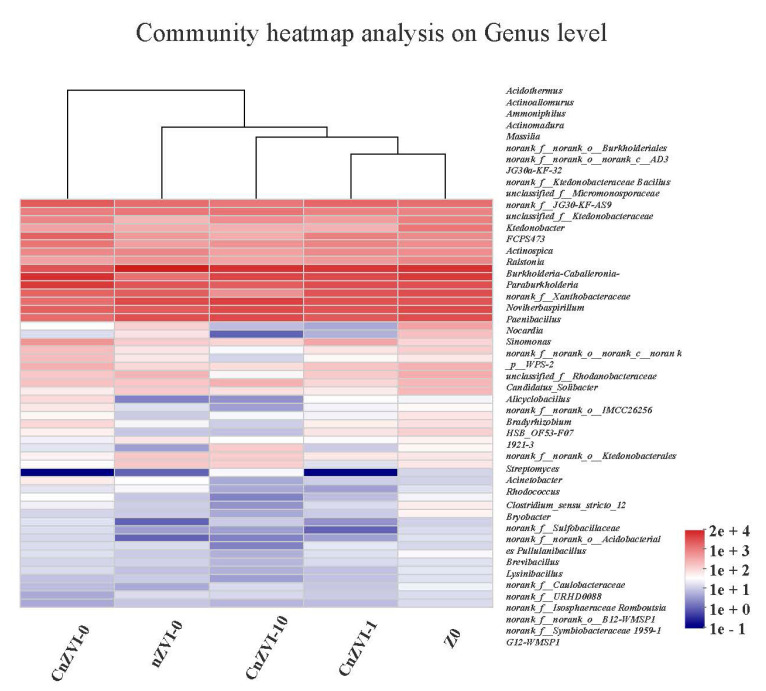
Hierarchical cluster analysis of microbial communities among the 5 samples. Different samples were clustered based on the complete linkage method. The color intensity of scale indicates the relative abundance of each genus.

From the clustering tree of the community heatmap analysis at genus level (Figure 8), we can see that Z0 and CnZVI-1 groups are the most similar, and the difference with the CnZVI-0 group is the largest. nZVI-0 and CnZVI-10 groups each have different biotoxicity. This indicates that CA concentration has a great influence on the distribution of microbial communities, and that a high concentration of CA is detrimental to the growth of microorganisms, while CMC has the effect of mitigating the biotoxicity of nZVI.

**Figure 9:**
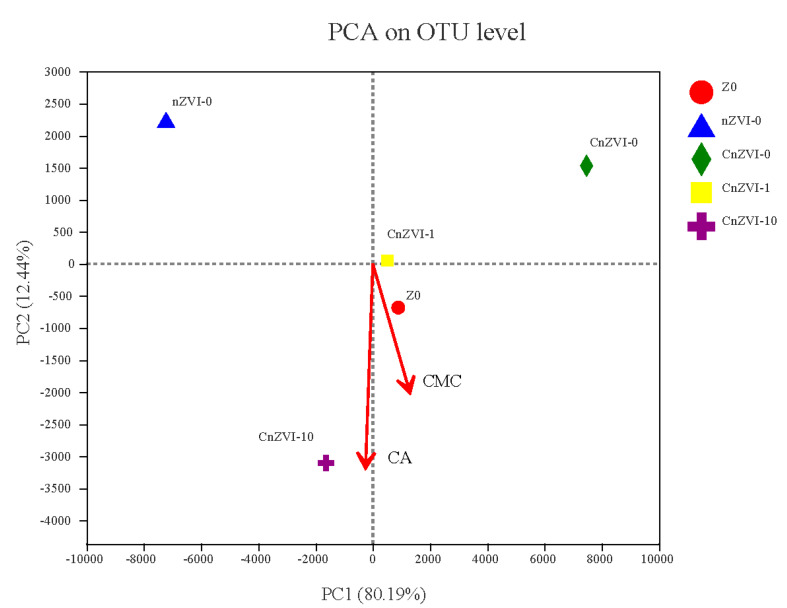
Output of the principal component analysis (PCA) among the 5 samples. Score plot of the first and second principal components (PC1, PC2, respectively).

Figure 9 shows the principal component analysis (PCA) of the OUT level. The distance between the samples in the figure reflects the degree of similarity between the samples. The closer the distance, the more similar the samples are, the farther the distance, and the more different the samples are from each other. The close distance between Z0 and CnZVI-1 indicates that the microbial community distributions of the two groups are similar. nZVI-0, CnZVI-0 and CnZVI-10 are located on the three edges of the plane, indicating that they are quite different. This may be because the CnZVI-0 group had the least biological toxicity towards stabilizing Cd, the nZVI-0 group had strong biological toxicity due to the absence of CMC coating, and the CnZVI-10 group made the soil acidic due to the high concentration of CA. The increase was not conducive to the growth of microorganisms. In Figure 9, the two vectors CA and CMC are considered environmental factors, where the subtypes are shown as points and the quantitative type is shown as a vector; the distance from the subtype environmental factor point to the sample point can indicate how much the sample is affected by the environmental factor; the distance from the sample point to the projection of the quantitative environmental factor vector indicates the size of the sample affected by the environmental factor.

### 3.8. The Interaction Mechanism between CA and nZVI under Cd Stress and the Response Mechanism of Microorganisms

**Figure 10:**
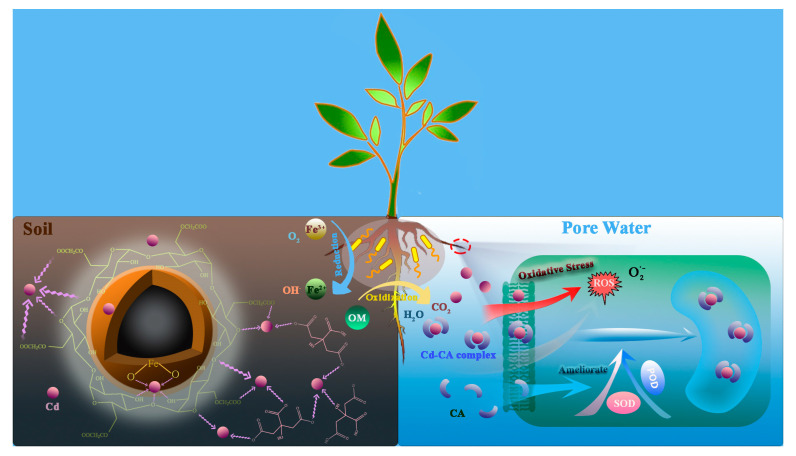
Proposed inner mechanisms for the nanomaterials’ remediation of Cd-polluted soil in the presence of root exudates and microflora.

When plants are subjected to Cd stress and the oxidation of nZVI to increase the pH of the rhizosphere soil, it will promote the plant roots to secrete more LMWOAs. This experiment uses CA as an example to simulate the rhizosphere soil environment, and the rhizosphere soil environment is divided into soil environment and pore water environment (Figure 10). CA (soil) and CnZVI-6 have a synergistic effect for complexation with the mobile Cd in the soil, thereby stabilizing Cd on clay minerals and reducing its biological effectiveness. The free Cd in the pore water will stimulate the oxidative stress response of plants, and produce a large number of free radicals, which will damage the plant cell structure. CA (water) can complex with the free Cd in the pore water to form a Cd–CA complex, and thus reducing the biological toxicity of Cd. At the same time, studies have shown that CA can promote the production of superoxide dismutase and peroxidase in plant cells, thereby slowing the oxidative stress response of plants under Cd stress. The content of CA (soil) and CA (water) is related to the total amount of CA present in soil and the physical and chemical properties of the soil, such as water–soil ratio, mineral composition, pH and OM. For the microorganisms around the rhizosphere, the OM in the soil is degraded into H_2_O and CO_2_, and the iron-reducing bacteria can reduce the Fe(Ⅲ) from oxidized Fe^0^ to Fe(Ⅱ).

## 4. Conclusions

In this study, the rhizosphere environment was simulated by adding CA to water and soil. Additionally, the experiment results indicated a plausible explanation for the phenomenon of zero-valent iron nanomaterials promoting both Cd stabilization in soil and plant uptake. In addition, the role of CA and the microorganisms was also investigated. It was concluded that CA could promote the immobilization of Cd by nZVI at a low concentration, thus reducing the bioavailability of Cd; when the concentration of CA was high, the immobilization effect of nZVI was weakened on the one hand. On the other hand, CA promoted the desorption of Cd and the generation of CA–Cd complexes, which reduced the biotoxicity and improved the bioavailability of Cd. The correlation between the soil properties (pH, OM and CEC) was also considered in this study. Additionally, the results indicated that OM was in a negative correlation with pH and CEC, and CEC was in a positive correlation with pH. Regarding the distribution of microbial communities, it was found that a high concentration of CA was not conducive to the growth of microorganisms, while the CMC had the effect of alleviating the biotoxicity of nZVI. In short, this is a small step forward for future investigations into nanomaterial applications in more complex and realistic rhizosphere environments.

## Data Availability

The data that support the findings of this study are available from the corresponding author upon reasonable request.

## Supplementary Materials

The following are available online at https://www.mdpi.com/article/10.3390/ijerph18115887/s1, Text S1: Nanoparticles Synthesis and Characterization, Text S2: Fractions of Cd in the soil samples, Text S3: Methods for the detection of soil physico–chemical properties, Text S4: High-throughput sequencing, Figure S1: SEM images of bare nZVI under (**A**) 1 μm scale and (**B**) 500 nm scale, and CnZVI-6 under (**C**) 1 μm scale and (**D**) 500 nm scale, Figure S2: XRD spectra of freshly prepared bare nZVI and CnZVI-6.
