# Supplementary material for: Remediation of Cd-Contaminated Soil by Modified Nanoscale Zero-Valent Iron: Role of Plant Root Exudates and Inner Mechanisms
Source: Int J Environ Res Public Health. 2021 May 30;18(11):5887. doi: 10.3390/ijerph18115887 (PMC8197846; doi:10.3390/ijerph18115887)
Supplement: Supplementary file 1 [file ijerph-18-05887-s001.zip › ijerph-1199574-supplementary.pdf]

Supplementary Materials

Remediation of Cd-Contaminated Soil by Modified Nanoscale Zero-Valent Iron: Role of Plant Root Exudates and Inner Mechanisms

Danlian Huang [1,2,*], Yunhe Yang [1,2], Rui Deng [1,2], Xiaomin Gong [3], Wei Zhou [1,2], Sha Chen [1,2], Bo Li [1,2] and Guangfu Wang [1,2]

[1] College of Environmental Science and Engineering, Hunan University, Changsha, Hunan 410082, China; yangyunhe@hnu.edu.cn (Y.Y.); dengrui703@hnu.edu.cn (R.D.); vzhou@hnu.edu.cn (W.Z.); sasa@hnu.edu.cn (S.C.); hndxlibo@hnu.edu.cn (B.L.); wangguangfu1113@163.com (G.W.)

[2] Key Laboratory of Environmental Biology and Pollution Control, Hunan University, Ministry of Education, Changsha, Hunan 410082, China

[3] College of Resources and Environment, Hunan Agricultural University, Changsha, Hunan 410128, China; gongxm2014@yeah.net

* Correspondence: huangdanlian1981@163.com

Text S1 Nanoparticles Synthesis and Characterization

The synthetic procedure was adapted from the experiments of Huang and Xue et al. [1-3]. Briefly, the nZVI and CMC-nZVI were synthesized through the reduction of ferrous sulfate by sodium borohydride at the BH_4^-/Fe^{2+} molar ratio of 2.0. And the entire process was carried out in solution under nitrogen atmosphere with mechanical stirring. The only difference was the absence or addition of various concentrations of CMC (0, 0.1, 0.15, 0.2, 0.25 wt%) to the solution. After synthesis, the nanoparticles were separated by magnetic force and then washed several times with deoxygenated deionized water (18.25MΩ cm, Barnstead D11911) and alcohol to remove impurities before being dried in a vacuum oven and stored in brown bottles filled with nitrogen.

Scaning Eectron Microscope (SEM) (Zeiss Gemini 300) was used to obtain the SEM images of nZVI and CMC-nZVI for observing their superficial morphological characteristics. X-ray diffraction (XRD) patterns of nZVI and CMC-nZVI were determined to analyze the mineralogy by a powder XRD system (Brucker, AXS D8 Advance). The diffraction angle is scanned from 5° to 90° at a rate of 2°/min.

Text S2 Fractions of Cd in the soil samples

The speciations of Cd in samples were measured by the modified sequential extraction method of European Community Bureau of Reference (BCR) [4,5]. The extractions were divided into four steps corresponding to four fractions separately. Acid extractable/exchangeable fraction (i.e. carbonates and the exchangeable fraction): for step 1, 40 mL of 0.11 M acetic acid was added to a centrifuge tube containing 0.5 g of sample and shaken for 16 h at room temperature. Subsequently, the supernatant was removed by

centrifugation for detection, while the solid residue was washed by centrifugation with deionized water which was then decanted for step 2. Reducible fraction (i.e. iron and manganese oxides fraction): 40 mL of freshly prepared hydroxylammonium chloride was added to the residue of step 1. The following steps were the same as above. Oxidizable fraction (i.e. organic and sulfide fraction): the residue from step 2 was treated by 10 mL of 8.8 M hydrogen peroxide twice. Digestion was carried out for 1 h at room temperature with occasional manual shaking, followed by an additional digestion for 1 h in a water bath at 85 ± 2°C until the mixture volume reduced to 2-3 mL. The previous step was repeated again and 50 mL of 1.0 M ammonium acetate was added to the residue in the tube, followed by 16 h of shaking at room temperature. Residual fraction (i.e. the metals in the crystalline lattice of primary and secondary minerals): the residue of step 3 was digested with aqua regia and hydrofluoric acid. All the above extracts were filtered through a 0.45 μm organic filter membrane and then measured by atomic absorption spectrometry.

Text S3 Methods for the detection of soil physicio-chemical properties

Soil pH was measured by diffusing 1 g of soil into water at a ratio of 1:5 w/w of soil to water and determined through inserting the electrode of a pH meter into the supernatant.

The method for the determination of soil OM content was obtained from the previous research, which was known as the potassium dichromate volumetric method-the dilution heating method [6]. The principle is to oxidize soil organic matter with a known concentration of potassium dichromate-sulfuric acid solution under external heating conditions. The OM value was calculated by the following equation:

$$OM(g/kg)=\frac{0.5\times(V_0\text{-}V)\times0.001\times3.0\times1.33}{M}\times1000\times1.724 \quad (1)$$

where V_0 is the $FeSO_4$ volume consumed by silicon dioxid of the blank, the V is the consumption of the $FeSO_4$ by the sample and the M is the weight of the soil sample.

The CEC value was determined by barium chloride - sulfuric acid enhanced exchange method method [4]. The principle is that the various cations present in the soil can be equivalently exchanged with the Ba^{2+} in the barium chloride ($BaCl_2$) solution. The Ba^{2+} exchanged into the soil are then exchanged out by strong electrolyte sulfuric acid (H_2SO_4) solution, and due to the generation of barium sulfate (Ba_2SO_4) precipitate, and the exchange and adsorption capacity of hydrogen ions is very strong, so that the exchange tends to be complete. Thus, by measuring the change of sulfuric acid content before and after the exchange reaction, the result of the CEC can be calculated using the equation as follows:

$$CEC\left(\frac{cmol}{kg}\right)=\frac{[(C_H\times50)\text{-}(C_N\times V_N)]\times100}{W_0K_2} \quad (2)$$

where C_H is the concentration of H_2SO_4 solution, C_N is the concentration of NaOH solution, V_N is the volume consumption of

NaOH solution, W_0 is the weight of the soil sample and K_2 represent the moisture content of the sample.

Text S4 High-throughput sequencing

The primers for amplification of bacterial 16S rRNA in the V4 region used in this experiment were 338 F (5′-ACTCCTACGGGAGGCAGCAG-3′) and 806 R (5′-GGACTACNVGGGTWTCTAA-3′) [7]. In this study, high-throughput sequencing was performed on the PCR products of specific fragments such as 16S rRNA functional genes to explore the evolution of soil microbial community after the above teatment, followed by investigating the inner mechanisms of simulated rhizosphere environment. Five samples, (Z0 (control group), nZVI_0 (nZVI only), CnZVI_0 (CnZVI only), CnZVI_1 (CnZVI + 50mg/L CA), and CnZVI_10 (CnZVI +500 mg/L CA)), were preincubated for 30 days and then outsourced to Shanghai Majorbio Bio-pharm Technology Co., Ltd, China for the high-throughput sequencing. The final data were obtained from the free online platform of Majorbio Cloud Platform (www.majorbio.com).

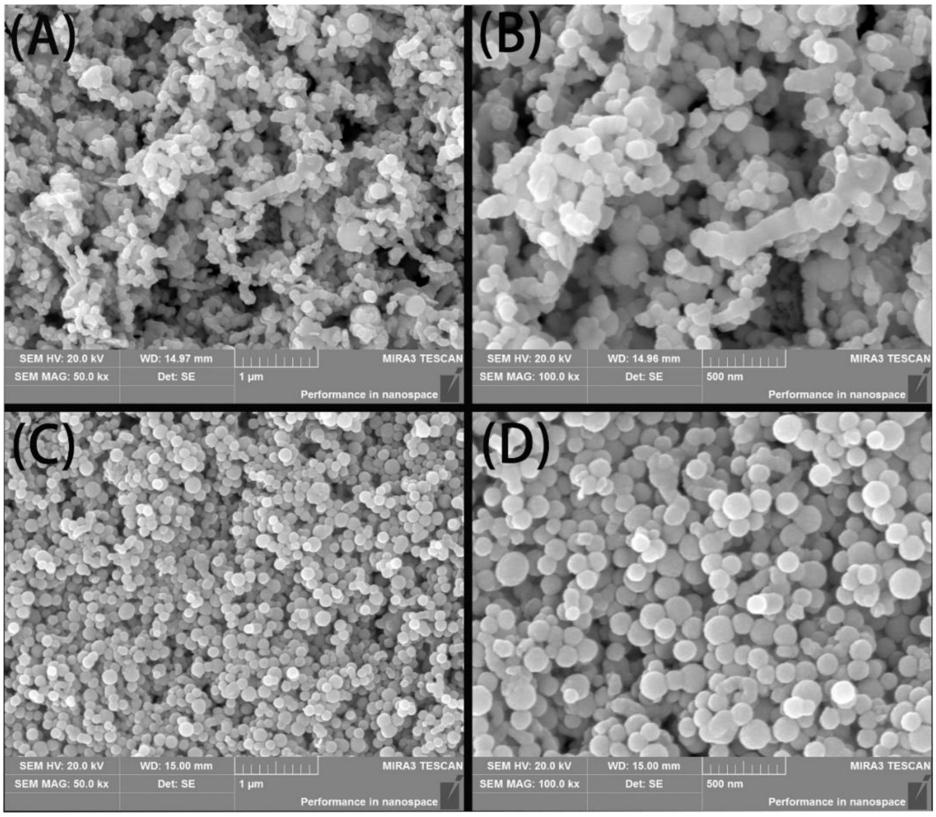

Figure S1. SEM images of bare nZVI under (A) 1 μm scale and (B) 500 nm scale, and CnZVI-6 under (C) 1 μm scale and (D) 500 nm scale.

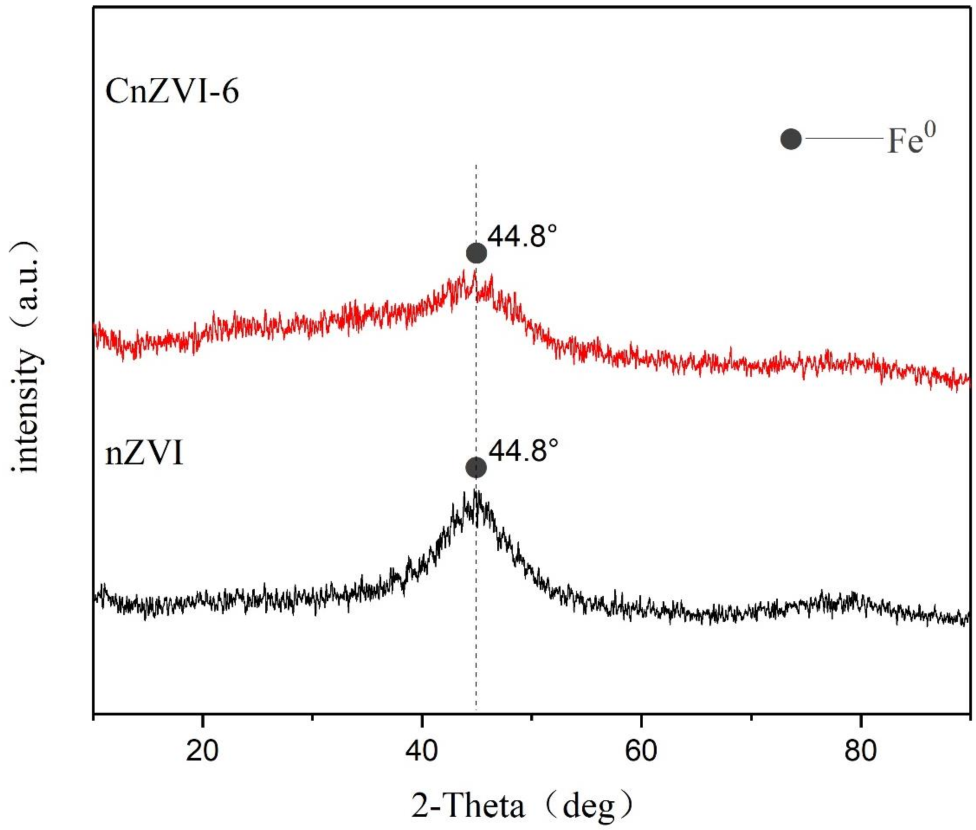

Figure S2. XRD spectra of freshly prepared bare nZVI and CnZVI-6.

Reference

1. Huang, D.; Hu, Z.; Peng, Z.; Zeng, G.; Chen, G.; Zhang, C.; Cheng, M.; Wan, J.; Wang, X.; Qin, X.J.J.o.E.M. Cadmium immobilization in river sediment using stabilized nanoscale zero-valent iron with enhanced transport by polysaccharide coating. **2018**, *210*, 191–200.
2. Huang, D.L.; Xue, W.J.; Zeng, G.M.; Wan, J.; Chen, G.M.; Huang, C.; Zhang, C.; Cheng, M.; Xu, P.A. Immobilization of Cd in river sediments by sodium alginate modified nanoscale zero-valent iron: Impact on enzyme activities and microbial community diversity. *Water Research* **2016**, *106*, 15–25, doi:10.1016/j.watres.2016.09.050.
3. Xue, W.; Huang, D.; Zeng, G.; Wan, J.; Zhang, C.; Xu, R.; Cheng, M.; Deng, R. Nanoscale zero-valent iron coated with rhamnolipid as an effective stabilizer for immobilization of Cd and Pb in river sediments. *Journal of Hazardous Materials* **2018**, *341*, 381–389, doi:https://doi.org/10.1016/j.jhazmat.2017.06.028.
4. Nanoremediation of cadmium contaminated river sediments: Microbial response and organic carbon changes. *Journal of Hazardous Materials* **2018**, *359*, 290–299, doi:https://doi.org/10.1016/j.jhazmat.2018.07.062.
5. Nemati, K.; Bakar, N.K.A.; Abas, M.R.; Sobhanzadeh, E. Speciation of heavy metals by modified BCR sequential extraction procedure in different depths of sediments from Sungai Buloh, Selangor, Malaysia. *Journal of Hazardous Materials* **2011**, *192*, 402–410, doi:https://doi.org/10.1016/j.jhazmat.2011.05.039.
6. Deng, R.; Huang, D.; Xue, W.; Lei, L.; Chen, S.; Zhou, C.; Liu, X.; Wen, X.; Li, B. Eco-friendly remediation for lead-contaminated riverine sediment by sodium lignin sulfonate stabilized nano-chlorapatite. *Chemical Engineering Journal* **2020**, *397*, 125396, doi:https://doi.org/10.1016/j.cej.2020.125396.
7. Ovreas, L.; Forney, L.; Daae, F.L.; Torsvik, V. Distribution of bacterioplankton in meromictic Lake Saelenvannet, as determined by denaturing gradient gel electrophoresis of PCR-amplified gene fragments coding for 16S rRNA. *Applied and environmental microbiology* **1997**, *63*, 3367–3373.
